# Environmental constraints can explain clutch size differences between urban and forest blue tits: Insights from an egg removal experiment

**DOI:** 10.1111/1365-2656.14171

**Published:** 2024-09-01

**Authors:** Mark D. Pitt, Norah S. S. Alhowiti, Claire J. Branston, Eugenio Carlon, Jelle J. Boonekamp, Davide M. Dominoni, Pablo Capilla‐Lasheras

**Affiliations:** ^1^ School of Biodiversity, One Health & Veterinary Medicine, Graham Kerr Building University of Glasgow Glasgow UK; ^2^ Biology Department, Faculty of Science University of Tabuk Tabuk Saudi Arabia; ^3^ School of Health and Life Sciences University of the West of Scotland Lanarkshire UK; ^4^ Department of Environmental Science and Policy Università Degli Studi di Milano Milan Italy; ^5^ Bird Migration Unit Swiss Ornithological Institute Sempach Switzerland

**Keywords:** adaptation, blue tit, breeding constraints, clutch size, urbanisation

## Abstract

1. Urban environments present novel ecological challenges to wild species. In birds, urban populations generally exhibit reduced clutch sizes compared to forest populations. However, whether smaller urban clutches are adaptive or a result of environmental constraints is unclear.
2. To investigate these two hypotheses, we quantified the ability of urban and non‐urban blue tits (*Cyanistes caeruleus*) to lay new eggs after an experimental manipulation aimed to increase egg production. We removed the first four eggs laid by urban and forest birds to test their ability to produce new eggs. If the urban environment imposes constraints on egg production, we predicted that urban birds would not lay new eggs. If the small clutches of urban birds are an adaptive response, we predicted they would lay new eggs to reach the optimal clutch size for the environment.
3. Consistent with the environmental constraint hypothesis, our results suggest that urban females do not lay new eggs to the same extent as forest birds following egg removal. Forest birds laid approximately two new eggs after our experimental manipulation, while urban birds laid approximately 0.36 new eggs following egg removal.
4. Our manipulation resulted in a brood reduction in the urban experimental nests, yet there was no difference in the number of fledged offspring between urban control and experimental nests. This suggests that females might be misjudging urban habitat quality and produce a clutch with more eggs than nestlings they can rear.
5. Overall, our results suggest that environmental constraints could limit the number of eggs that urban females lay, generating urban versus non‐urban differences in this trait.

## Graphical abstract

In birds, urban populations generally exhibit reduced clutch sizes compared to forest populations. Following an egg removal manipulation that aimed to increase egg production, urban birds did not lay as many new eggs as forest birds, suggesting that environmental constraints could limit the number of eggs that urban females lay.

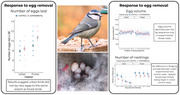

## 1 INTRODUCTION

Urban land coverage is expanding globally and is projected to amount to 1.6 million km^2^ by 2100 (Gao & O'Neill, 2020; Liu et al., 2018). Urban environments have a distinct set of ecological features, characterised by increased ambient temperatures (Kim, 1992), a high abundance of non‐native species (McKinney, 2008), increased habitat fragmentation (Van Bohemen, 1998), increased pollution (Dorado‐Correa et al., 2016; Isaksson, 2018) and changes in the quality and availability of food (Fehlmann et al., 2021; Fenoglio et al., 2021; Jensen et al., 2022). Urban environmental conditions create novel ecological pressures to which species might not be well adapted, potentially compromising the persistence of wildlife globally. Understanding the mechanisms behind biological changes associated with urbanisation is crucial to determine the current and future impact of urbanisation on population dynamics and biodiversity.

In birds, recent meta‐analyses suggest that among the phenotypic changes that occur in urban habitats, reductions in reproductive investment (e.g. clutch size) are pervasive and taxonomically widespread (Capilla‐Lasheras et al., 2022; Chamberlain et al., 2009; Sepp et al., 2018). For example, in great tits (*Parus major*) and blue tits (*Cyanistes caeruleus*), the clutch sizes of urban breeding birds tend to be 10%–20% smaller than those nesting in non‐urban habitats (Branston et al., 2021; Glądalski et al., 2017). However, the explanation for these differences in reproductive investment remains unclear; the small clutches of urban birds might represent a constraint imposed upon females by the urban environment when laying or reflect an adaptation to urban living.

Urban environments could impose constraints on birds during egg production, limiting the female's ability to invest in a larger clutch. In this scenario, the clutch size birds produce would be constrained by the quality and quantity of available resources (constraint hypothesis; Mänd et al., 2007). Egg production is metabolically demanding, with the energy required for egg‐laying ranging between 13% and 41% above the basal metabolic rate in passerines (Carey, 1996). In small passerines that produce large clutches, the resources required for egg production far exceed what females can store endogenously (Meijer & Drent, 1999; Perrins, 1996). Therefore, birds need to source the energy and nutrients for egg formation (including proteins, antioxidants, omega‐3 polyunsaturated fatty acids and calcium), from their daily diet when laying, with invertebrates (e.g. spiders, caterpillars) being the most nutrient‐rich food items (Andersson et al., 2015; Biard et al., 2005; Graveland & Drent, 1997). Habitat fragmentation, non‐native plant species, pollution and increased pesticide use in urban areas reduce the quality and quantity of invertebrate prey (Fenoglio et al., 2021; Jensen et al., 2022). Urban birds may attempt to compensate for the reduced availability of invertebrates by exploiting the abundant and predictable human‐provisioned food from refuse and bird feeders (Jarrett et al., 2020). However, anthropogenic food, despite being energy‐rich, is nutritionally poor and contains limited proteins, antioxidants and omega‐3 polyunsaturated fatty acids (Isaksson, 2018; Toledo et al., 2016). Thus, if nutrient‐constrained, urban birds may be laying as many eggs as they can produce given the low quality or quantity of resources in the urban environment. Under the constraint hypothesis, the smaller clutch size of urban birds compared to non‐urban birds would be a result of an environmental limitation.

Alternatively, the small clutches of urban birds may be an adaptive response to urban living (i.e. genetic change in response to selection). In this scenario, rather than being constrained by the available resources when laying, the smaller clutch sizes of urban birds would be the result of selection, with any deviation from this observed clutch size resulting in fewer offspring being recruited into the population (adaptive hypothesis; Lack, 1947, 1954). Previous research reveals that urban birds have smaller broods and fledge fewer offspring than their forest counterparts (Capilla‐Lasheras et al., 2022; Chamberlain et al., 2009). Urban birds may be producing small clutches as an adaptive response, laying fewer eggs to match the smaller number of young that they can adequately provision and rear to independence in the urban habitat (Senar et al., 2021). Small clutch sizes could allow urban parents to invest more resources into fewer nestlings, reducing sibling competition and thereby maximising the number of offspring recruited (Sepp et al., 2018).

Previous studies have undertaken egg removal experiments to understand the sources of variation in clutch size (Mänd et al., 2007; Monaghan & Nager, 1997; Nager et al., 2001; Visser & Lessells, 2001). In the absence of environmental constraints, females lay new eggs following egg removal to keep as close as possible to the optimal clutch size for the habitat (Haywood, 1993b; Monaghan & Nager, 1997). If egg production is environmentally constrained during laying, then females produce fewer or no new eggs following the egg removal manipulation; when new eggs are produced, their size may be reduced (Mänd et al., 2007; Visser & Lessells, 2001).

Here, we investigate whether differences in clutch size between an urban and a non‐urban population of blue tits are explained by environmental constraints or whether they represent an adaptive strategy. Blue tits are a cavity‐nesting forest species but have readily colonised urban environments (Stenning, 2018). Due to their prevalence in urban areas, willingness to use nest boxes and ability to tolerate human disturbance, blue tits are an ideal study system for investigating how urbanisation influences reproductive decisions. If not environmentally constrained, blue tits lay one new egg in response to the removal of the first two laid eggs (Haywood, 1993a; Stenning, 2018). Thus, the number of eggs that females produce can be experimentally manipulated by removing eggs from the clutch before the female initiates incubation.

During one breeding season, we removed the first four eggs laid in urban and non‐urban blue tit nests and observed how this manipulation affected the total number and size of eggs laid. Subsequently, we examined how egg removal influenced offspring quality at hatching and nestling survival to fledging. If differences in clutch size between habitats are caused by environmental constraints on egg production, we predict that, following the egg removal manipulation, urban females should lay fewer, or smaller, eggs than non‐urban females (as environmental constraints could also be reflected in egg size). If this was the case, we predict that nestling survival to fledging should be higher in experimental nests than in control nests because the resulting reduction in brood size should lead to lower intra‐brood competition (Nicolaus et al., 2009). If egg production in urban females is not constrained by the environment (and, possibly, reflects an adaptation to urban conditions), we predict that urban and non‐urban females should lay a similar number of new eggs of equal size following the egg removal manipulation. We would also predict no difference in hatchling body mass, nestling survival to fledging and nestling body mass between treatment groups.

## 2 METHODS

### 2.1 Study populations

We monitored one urban and one non‐urban nest box population of blue tits in Scotland from 1st April to 30th June (woodcrete nest box: 260H × 170W × 180D mm, hole diameter: 32 mm). The urban population resides in a city park in Glasgow (Kelvingrove Park, coordinates = [55.869, −4.2851]) and contains open land, trees and small shrubs, with 40% of the tree species being non‐native (Table S1). The non‐urban (‘forest’ hereafter) population resides in an ancient native oak woodland 40 km north of Glasgow (Scottish Centre for Ecology and the Natural Environment (SCENE), coordinates = [56.129, −4.6145]). The dominant tree species at SCENE are native broadleaved trees, with only 2% of the trees comprising non‐native species (Table S1; Capilla‐Lasheras et al., 2017; Pollock et al., 2017).

### 2.2 Experimental design

**FIGURE 1:**
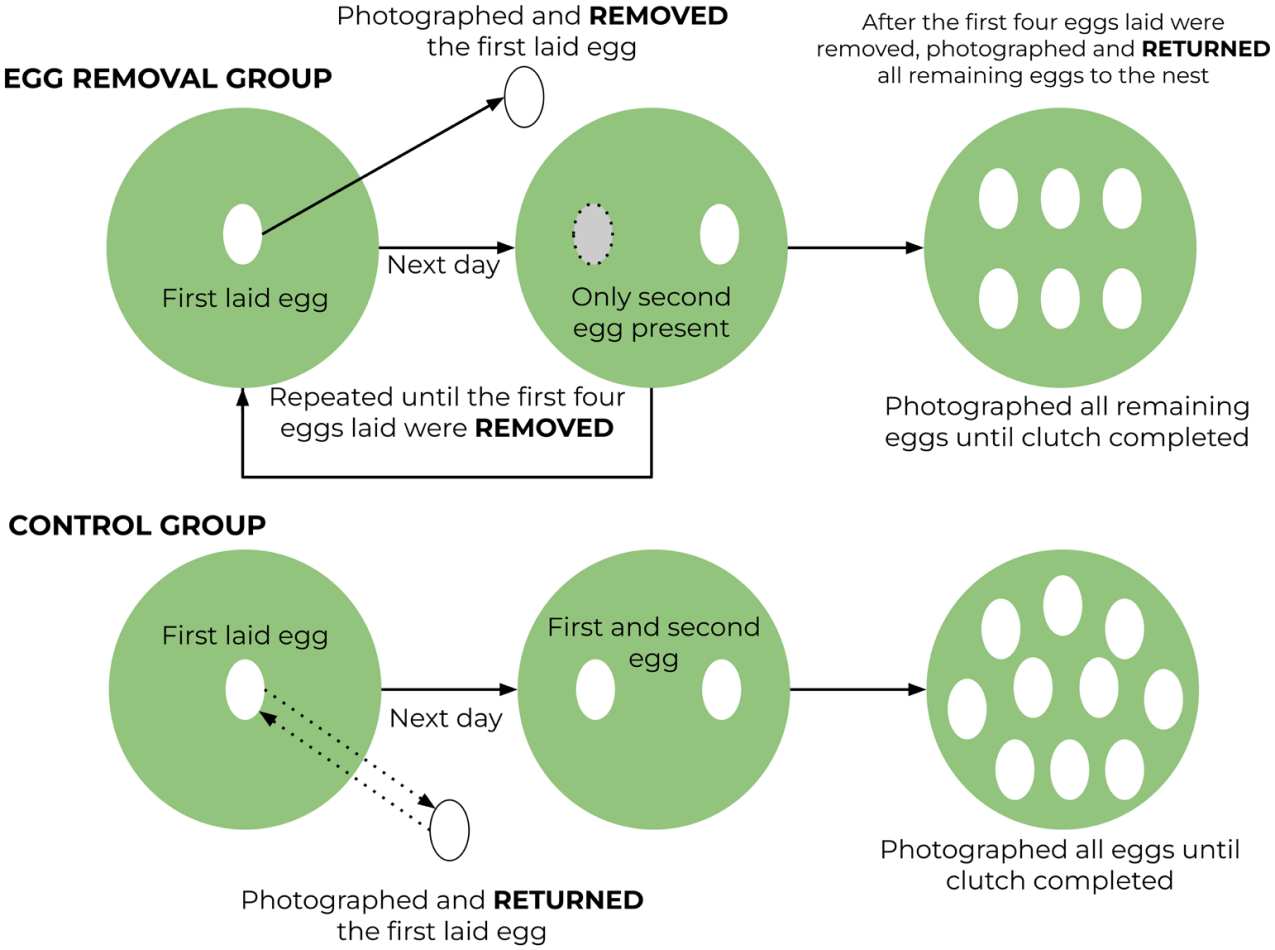
Overview of experimental design, showing the procedure for each treatment group. The first four eggs were photographed, marked and removed from experimental egg removal nests on the day they were laid. After the first four eggs laid were removed, we photographed and returned all remaining eggs to the nest. No eggs were removed from control nests, with eggs only being handled when photographed and marked.

From 1 April 2022, we visited nest‐boxes twice weekly to monitor nest‐building activity. We increased the frequency of visits to every two days once the blue tits started lining the nest‐cup to ensure we accurately recorded the first egg laying date. We visited nests after 11:00 to ensure sufficient time for the blue tit to lay (in passerines, most egg‐laying occurs shortly after sunrise; McMaster et al., 2004). Within the same habitat, once a new clutch was detected, it was alternatively assigned to the control or experimental group, following a 1:1 ratio to reduce any difference in phenology between treatment groups. For nests included in the study, we found no difference in the first egg laying date between treatment groups (*χ* ^2^_df=1_ = 0.118, *p* = 0.732). Once a new egg was found, we marked them using a non‐soluble marker pen, with a number corresponding to the eggs position in the laying sequence. We photographed every egg (used to calculate egg volume; details below) in both control and experimental nests. Control nests had no eggs removed, with the photographing and marking of eggs being the only time we disturbed control females during egg production (Figure 1). In experimental nests, we removed the first four eggs from the nest on the morning each egg was laid. Eggs removed from the experimental nests were also weighed (± 0.01 g) using digital scales. We did not return any of the removed eggs back to the clutches of experimental females; they were collected and frozen for future studies. When the female stopped laying for two days, and the eggs were warm to touch, we assumed the clutch was complete, and incubation had commenced (Womack et al., 2023). We considered the experimental manipulation successfully completed when females laid eggs past the egg removal phase, which occurred in 66 clutches (29 in the urban habitat [14 control and 15 egg removal clutches]; 37 in the forest habitat [20 control and 17 egg removal clutches]). Out of those, we could not confirm clutch completion in five clutches (two in the urban habitat and three in the forest). These five clutches are only included in the egg volume analysis. An additional two clutches, which were completed (i.e. incubation started), were abandoned before the eggs hatched (both clutches in the forest) and are not included in our analyses of nestling traits. Thirteen days after clutch completion, we started nest box visits every two days to record hatch date. Nestlings were cross fostered between habitats on day two of their life to disentangle urban environmental effects from maternal/genetic effects on weight on Day six, 12 and nestling survival (see Supplementary methods in Appendix S1 for details). We included habitat of rearing and habitat of hatching in our models of nestling body mass on Day six and 12. Six days after hatching, nestlings were fitted with a unique British Trust for Ornithology (BTO) metal ring. We weighed nestlings to the nearest 0.1 g on Day two (before cross fostering manipulation), six and 12 after hatching. Nest‐boxes were checked >21 days after hatching to record dead and fledged nestlings. All work involving nest disturbance, egg removal and cross‐fostering were covered by the licence 207317 issued by NatureScot to Davide M. Dominoni. Permission for bird ringing was granted by the British Trust for Ornithology, with licences to Davide M. Dominoni (permit number: C6822) and Claire J. Branston (permit number: C6271).

### 2.3 Egg volume measurements

All eggs in control and experimental clutches (both removed and not removed eggs) were treated in the same way. We used egg volume as a proxy of egg size, which reflects the level of pre‐natal investment into the young before incubation and may dictate offspring quality at hatching (Krist, 2011). We used an Olympus TG‐6 digital camera to photograph eggs in the field on a 20 × 20 cm measuring chart at a 90° angle to the egg's long axis. There were no adjustments to lens distortion. We photographed each egg four times, rotating the egg along its long axis between photographs. To calculate egg volume, we used IMAGEJ and the egg measurement plugin (Troscianko, 2014). Using the multipoint selection tool in IMAGEJ, we selected 12 anchor points around the edge of the eggs surface. For each egg, we calculated volume (mm^3^) from three separate images. All images were analysed by a single observer (MP) to minimise the risk of between‐observer variation. In total, 1160 images of 386 eggs were analysed. We used the R package *rptR v.0.9.22* (Stoffel et al., 2017) to quantify the repeatability of the individual volume measurements (see Supplementary methods in Appendix S1). The repeatability of egg volume between the three images of a single egg was low (repeatability [95% Confidence Interval ‘CI’] = 0.307 [0.237, 0.406]). However, individual females exhibited consistent egg size within clutches (repeatability [95% CI] = 0.578 [0.452, 0.667]) and mean egg volume across the three images for a single egg was highly correlated with egg mass (using removed eggs, *N* = 83 eggs, Pearson's correlation coefficient *r* [95% CI] = 0.743 [0.627, 0.826]), suggesting our measurement of egg volume captured biologically relevant variation. In all subsequent analyses of egg volume, we used each of the three volume measurements per egg as the response variable (rather than the mean volume per egg) and included egg ID (the individual identifier for each egg) as a random effect.

### 2.4 Data analysis

#### 2.4.1 General modelling procedure

We performed all statistical analysis and data visualisation in RStudio 2023.09.1 + 494 using R v.4.3.2 (R Core Team, 2022). We used the R packages lme4 (v.1.1.34; Bates et al., 2015) to build generalised linear mixed models (GLMM) to explain variation in the investigated reproductive traits (details below). The R package glmmTMB (v.1.1.7; Brooks et al., 2017) was employed to fit Conway–Maxwell–Poisson GLMs (details below) and binomial GLMMs. We built a full model for each reproductive trait, containing all explanatory variables and interactions hypothesised to explain variation in that trait. Wherever interaction effects were fitted, the main effect predictors of the interaction were also included as single‐effect predictors. We assessed the statistical importance of predictors using likelihood ratio tests. We did not apply model simplification of single effect predictors beyond the removal of non‐significant interaction effects (including quadratic terms) to ease the interpretation of single effect coefficients. In all models, date (either first egg laying date or hatching date) was expressed as the number of days since the 1st of January. Date variables were fitted as both a quadratic and linear term before quadratic terms were dropped from all the models if not significant. We used the R package DHARMA (v.0.10.4; Lüdecke et al., 2021) to assess model residuals. We used the package ggplot2 (v.3.4.2) to visualise model predictions and raw data; and the packages gt (v.0.9.0; Iannone et al., 2023) and gtsummary (v.1.7.2; Sjoberg et al., 2021) to produce model tables.

#### 2.4.2 Statistical models

##### Total number of eggs laid

We first used a Poisson GLM to explain variation in the total number of eggs laid by a female (including those that were removed as part of the experiment). However, model residuals were heavily under‐dispersed, and they did not follow the expected theoretical distribution. We, therefore, used a Conway–Maxwell–Poisson GLM and confirmed that the residuals of this model followed model assumptions. Conway–Maxwell–Poisson distribution is a two‐parameter distribution where mean and variance can be both quantified (Shmueli et al., 2005). This model used a log‐link function as in standard Poisson models. This model included, as fixed effect predictors, first egg laying date (mean‐centred), treatment group (two‐level factor, ‘egg removal’ vs. ‘control’) and habitat (two‐level factor, ‘urban’ vs. ‘forest’). The model also included the habitat × treatment group interaction. A total of 61 clutches were included in the analyses, including all clutches in which the experimental manipulation was completed successfully (see above). We then ran two separate within‐habitat models, one for the urban habitat and one for the forest habitat, including first egg laying date (mean‐centred) and treatment group as fixed effects.

##### Egg volume

To determine whether there were differences in egg volume between habitats and treatment groups, we built a Gaussian GLMM with egg volume as a response variable. As fixed effect predictors, we included habitat, treatment group, position of the egg in the lay sequence (fitted as a continuous variable), the total number of eggs laid by each female (mean‐centred), and egg‐laying date (the exact day each egg was laid; mean‐centred). This model also included the three‐way interaction between the position of the egg in the lay sequence, habitat and treatment group (as well as all the second‐order interactions between those terms). Clutch ID (a 59‐level factor) and egg ID (a 386‐level factor) were included as random effect intercepts (that also accounted for the nested nature of the data [i.e. eggs were observed within clutches]). We only included in the analysis eggs for which their laying date was accurately recorded. This analysis included 386 eggs from 59 clutches.

##### Hatchling and nestling body mass

We used a GLMM to determine whether egg removal differentially affected nestling body mass between habitats two days after hatching (*n* = 346 nestlings weighed on day two of their lives). As previous research suggests there may be a relationship between egg size and hatchling body mass; the habitat or treatment group with the largest eggs should also produce heavier hatchlings. We included nestling body mass two days after hatching as the response variable in a linear mixed model with habitat, treatment group, number of siblings (mean‐centred), hatch date (mean‐centred), time of day of the weight measurement (two‐level factor: ‘morning’ or ‘afternoon’), and the two‐way interaction between habitat and treatment group as fixed effects. Clutch ID (a 50‐level factor) was included as a random effect. Using linear mixed models with a similar fixed and random effect structure, we investigated variation in cross‐fostered nestling body mass on Day six and 12 after hatching (see Supplementary methods and results in Appendix S1; Tables S8 and S9).

##### Nestling survival to fledging

To investigate whether our experimental manipulation affected nesting survival to fledging, we ran two complementary analyses. First, we fitted a binomial GLMM with the proportion of alive nestlings at a given time point (i.e. nestling age: 4‐level factor: ‘Day 2’, ‘6’, ‘12’ or ‘fledged’) as the response variable (i.e. number of nestlings alive over clutch size). In this model, we included as fixed effects, egg removal treatment group, nestling age and the three‐way interaction between nestling age, habitat and treatment group (as well as the lower order interactions between these terms). Clutch ID (a 48‐level factor) was included as a random effect intercept. Second, we fitted a binomial GLMM where the response variable represented whether an individual nestling had survived or not to fledging. This model included as fixed effects, hatch date (days after January 1; mean‐centred), weight on day 2, egg removal treatment group, habitat of rearing, habitat of hatching and the two‐way interaction between habitat of rearing and treatment group. Nest box of hatching (a 48‐level factor) and nest box of rearing (a 47‐level factor) were included as a random effect intercepts.

## 3 RESULTS

### 3.1 Experimental effect of egg removal on the total number of eggs laid

Urban experimental females laid, on average, 8.36 eggs (standard error [SE] ± 0.487 eggs; *n* = 14 clutches) compared to 8.00 eggs (SE ± 0.358 eggs; *n* = 13 clutches) in urban control clutches. In the forest, experimental females laid, on average, 11.40 eggs (SE ± 0.491 eggs; *n* = 16 clutches) compared to 9.44 eggs (SE ± 0.414 eggs; *n* = 18 clutches) in forest control clutches. When looking at the average clutch sizes following egg removal, urban experimental nests contained 4.36 eggs (standard error [SE] ± 0.487 eggs; *n* = 14 clutches) compared to 8.00 (SE ± 0.358 eggs; *n* = 13 clutches) eggs in the urban control clutches. Meanwhile, forest experimental nests contained 7.44 eggs (SE ± 0.491 eggs; *n* = 16 clutches), while forest control nests contained 9.44 eggs (SE ± 0.414 eggs; *n* = 18 clutches).

**FIGURE 2:**
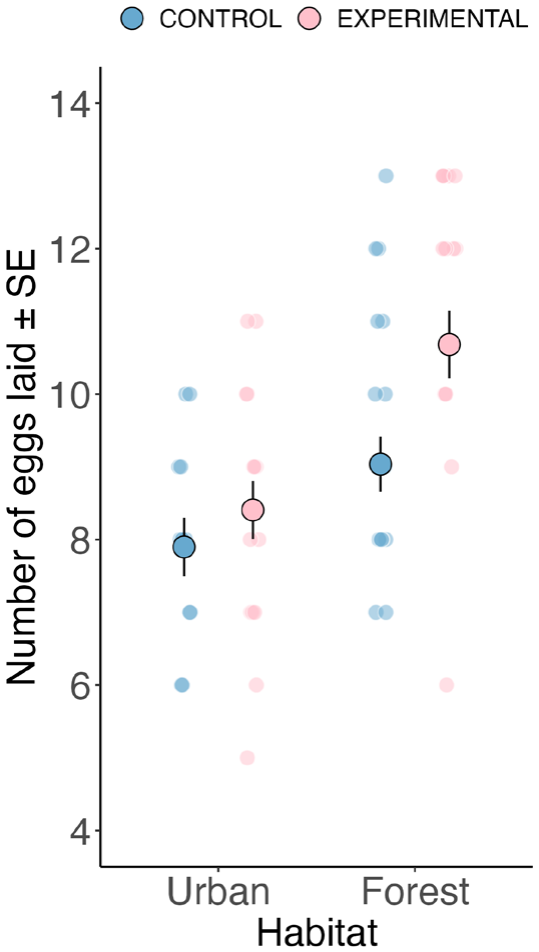
The effect of egg removal and habitat on the total number of eggs laid per female. Large dots represent model predictions ± one standard error (SE). Small dots are the raw data points.

A formal analysis of this data set revealed no statistical difference between habitats in the effect of egg removal on the total number of eggs that females laid (habitat × treatment group interaction, *χ* ^2^_df=1_ = 1.366, *p* = 0.243; Table S2). However, numerically (see above) and visually (Figure 2), there appeared to be a difference in the number of new eggs laid by manipulated females between the urban and forest habitat, suggesting that the lack of formal statistical support of the interaction might have been due to a low sample size. Creating two separate models, one for the urban and one for the forest habitat yielded a significant experimental effect in the forest (i.e. experimental forest females laid significantly more eggs than control forest females; *χ* ^2^_df=1_ = 7.696, *p* = 0.006; Table S3) but not in the urban habitat (*χ* ^2^_df=1_ = 0.713, *p* = 0.398; Table S4).

### 3.2 Experimental effect of egg removal on egg volume

**FIGURE 3:**
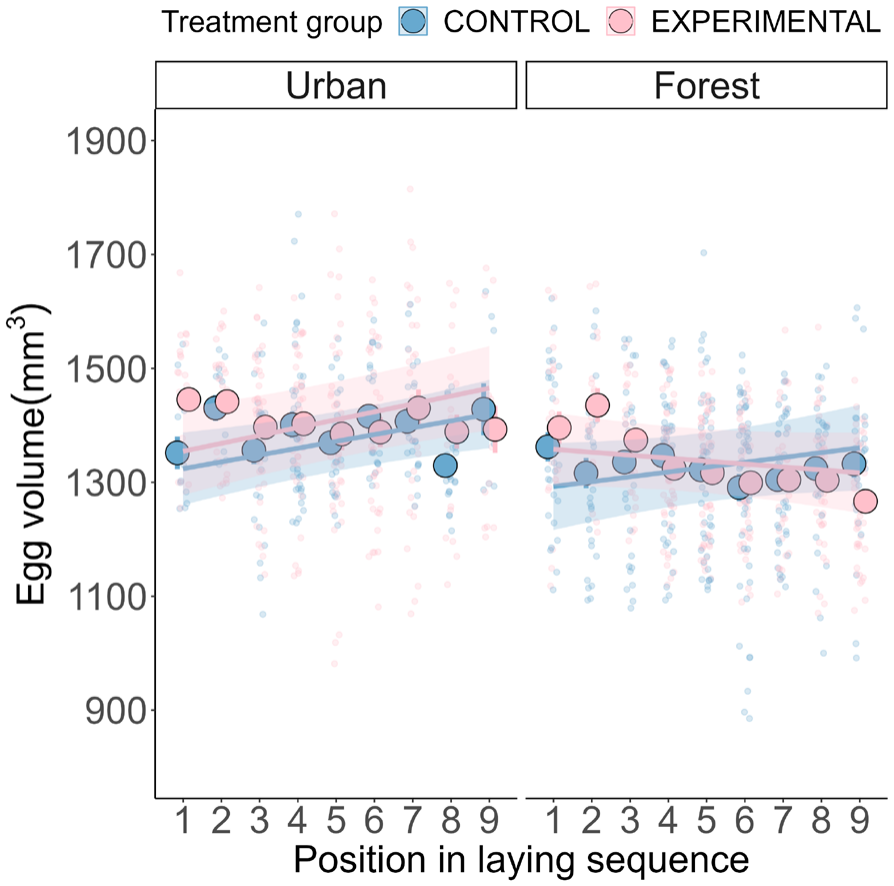
The effect of experimental egg removal and habitat on egg volume over the laying sequence. Large dots represent raw means ± SE. Solid lines and shaded area represent model predictions ±95% confidence intervals. Small dots are the raw data points.

In all treatment groups, egg volume slightly increased along the laying sequence, except for experimental forest females whose egg volume decreased along the laying sequence (three‐way interaction between laying sequence, habitat and treatment group, *χ* ^2^_df=1_ = 3.941, *p* = 0.047; Table S5; Figure 3). Egg volume increased throughout the laying sequence in both experimental (estimate ± SE = 13.69 ± 4.82 mm^3^/egg position) and control broods (estimate ± SE = 11.99 ± 5.80 mm^3^/egg position) in the urban habitat (with no difference in slope across groups: post hoc test, difference in control vs. experiment slopes = −1.87, *t*‐ratio = −0.300, df = 337, *p* = 0.764; Figure 3). However, while egg volume increased along the laying sequence in control forest broods (estimate ± SE = 8.51 ± 4.26 mm^3^/egg position), it decreased in experimental broods (estimate ± SE = −5.18 ± 4.37 mm^3^/egg position; post hoc test, difference in control versus experiment slopes = 13.69, *t*‐ratio = 2.93, df = 331, *p* = 0.004; Figure 3). We also found a seasonal effect on egg volume: the later an egg was laid in the season, the lower its volume (*χ* ^2^_df=1_ = 13.165, *p* < 0.001; Table S5).

### 3.3 Experimental effect of egg removal on nestling body mass

There was no differential effect of the egg removal on nestling body mass two days after hatching between habitats (treatment group × habitat interaction, *χ* ^2^_df=1_ = 0.032, *p* = 0.857). Nestlings did not differ in their weight on Day two between treatment groups or habitats (Table S6). Number of siblings and hatching date did not explain variation in nestling weight on Day two (Table S6). Experimental egg removal had no effect on nestling weight on Day six or 12 (Supplementary results in Appendix S1; Tables S7 and S8); the urban environment had a strong negative effect on the weight of 6‐ and 12‐day‐old nestlings (Supplementary results in Appendix S1; Tables S7 and S8).

#### 3.3.1 The effect of egg removal on nestling survival between habitats

Two days after hatching, urban experimental nests contained, on average, 4.11 nestlings (standard error [SE] ± 0.564 nestlings; *n* = nine broods) compared to 7.00 nestlings (standard error [SE] ± 0.618 nestlings; *n* = 11 broods) in urban control nests. However, the urban treatment groups fledged a similar number of offspring, with urban experimental nests fledging, on average, 1.89 offspring (standard error [SE] ± 0.655 offspring; *n* = nine broods) compared to 2.09 offspring (standard error [SE] ± 0.707 offspring; *n* = 11 broods) in urban control nests. Two days after hatching in the forest, experimental nests contained 6.71 nestlings (standard error [SE] ± 0.549 nestlings; *n* = 14 broods) compared to 8.79 nestlings (standard error [SE] ± 0.536 nestlings; *n* = 14 broods) in control nests. Forest experimental nests fledged, on average, 5.46 offspring (standard error [SE] ± 0.863 offspring; *n* = 14 broods) compared to 7.86 offspring (standard error [SE] ± 0.895 offspring; *n* = 14 broods) that fledged in forest control nests.

**FIGURE 4:**
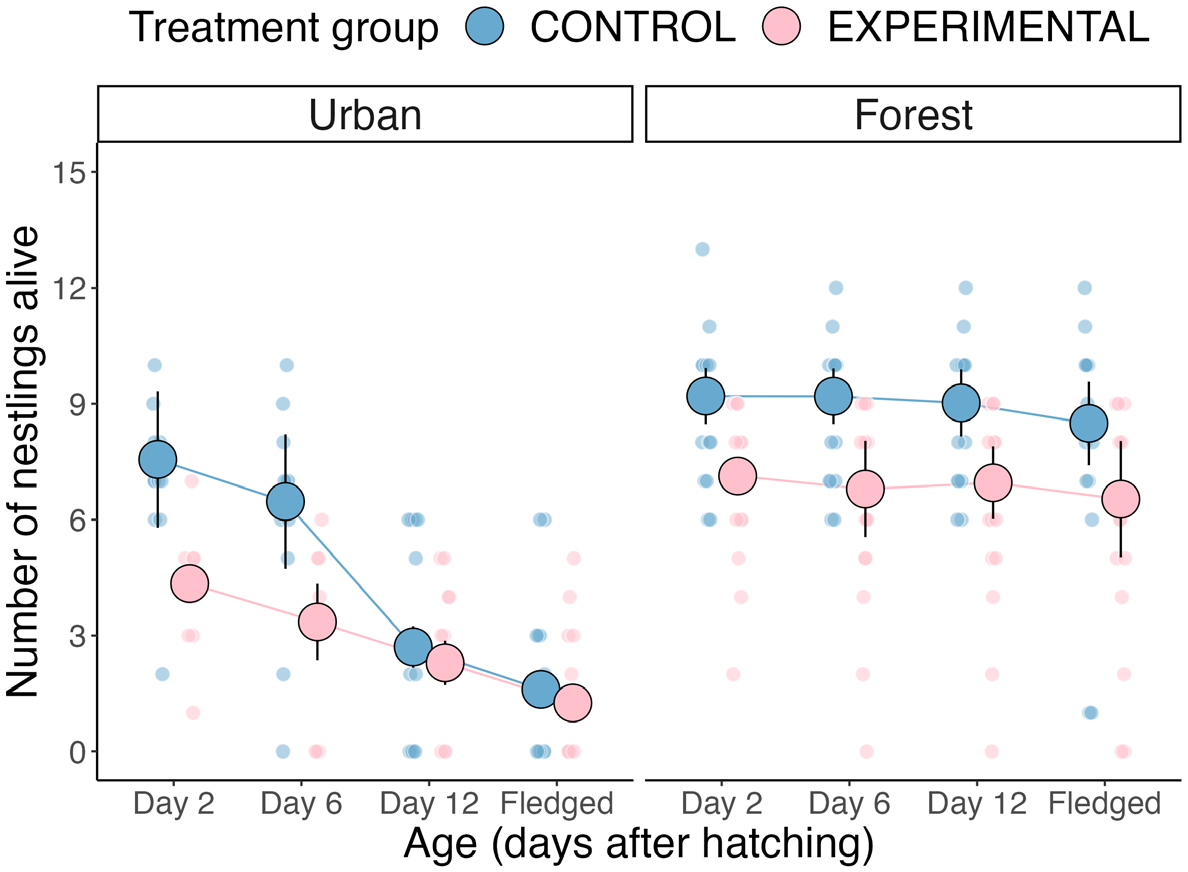
The effect of experimental egg removal and habitat on the number of nestlings. Egg removal treatment caused experimental forest females to have slightly smaller brood sizes, this effect stayed consistent throughout the breeding period. In urban broods, egg removal caused a larger decrease in brood size (due to the lack of compensatory egg laying); however, both experimental and control broods fledged a similar number of nestlings due to increased nestling mortality in control (i.e. larger) broods. Large dots and associated bars represent model predictions ± one standard error (SE). Small dots represent raw data points.

A formal analysis of this data set found that, while control broods in the forest were larger than experimental broods at each time point, in the urban habitat, control broods were larger than experimental broods only two and six days after hatching (Table S9; Figure 4). On Day 12 and at fledging, there was no difference between treatment groups in the number of nestlings alive in the urban habitat (Table S9; Figure 4). There was no difference in nestling survival probability between treatment groups in any environment (Table S10). However, heavier nestlings on Day two had higher survival probability (Table S10) and forest‐reared nestlings had higher survival probability than urban‐reared nestlings (Table S10). Habitat of hatching and hatch date did not predict individual nestling survival probability (Table S10).

## 4 DISCUSSION

Urban passerine birds consistently show reduced investment in their clutch size compared to their forest counterparts (Capilla‐Lasheras et al., 2022; Chamberlain et al., 2009; Sepp et al., 2018), but whether this is a constraint or adaptation to the urban environment has remained unclear. To test if the smaller clutches of urban birds reflected constraints imposed upon females by the urban environment or whether this reduction might represent an adaptation to urban conditions, we investigated the ability of females to lay new eggs following an egg removal manipulation in both urban and forest habitats. In line with the constraint hypothesis, our findings tentatively suggest that urban birds did not lay new eggs to the same extent as forest females. If urban blue tits were not constrained in some way during egg production, then urban females should have laid new eggs to compensate for the egg removal manipulation and maintain an optimal clutch size (Mänd et al., 2007; Visser & Lessells, 2001). This result needs to be cautiously interpreted as evidence came from habitat‐specific models. However, our findings based on the experimental effect size across habitats represent novel evidence for potential differences in environmental constraints on egg production between urban and non‐urban habitats. These constraints could contribute towards explaining why urban birds generally produce smaller clutches than their non‐urban counterparts, a pattern observed across many unrelated bird species (Capilla‐Lasheras et al., 2022; Chamberlain et al., 2009). Egg volume increased over the lay sequence for both urban treatment groups and control forest nests but declined over the lay sequence in experimental forest nests. Since urban females did not lay as many new eggs after egg removal when compared to forest birds, their final clutch size (and subsequent brood size) was reduced compared to urban control clutches. This created an unintentional artificial brood reduction that was stronger in the urban habitat than in the forest. Despite the brood size being approximately four nestlings higher in control than in experimental broods in the urban habitat on Day two post‐hatching, both treatment groups in the urban habitat fledged the same number of nestlings (Figure 4). This finding suggests that control females laid at least four eggs more than what they could successfully rear given the environmental conditions they experienced during our study. Below, we discuss alternative explanations of our findings and the extent to which they support our main hypotheses, including the limitations of this research and future directions to further understand the evolution of the optimal offspring number in anthropogenic environments.

### 4.1 Environmental constraints on egg production

Our data suggest that environmental constraints on egg production could be increased in urban areas. There is evidence suggesting that resources available to urban birds during egg laying could be of lower quality than in non‐urban areas (Jarrett et al., 2020; Pollock et al., 2017). Urban blue tits may have not responded to our egg removal manipulation due to being constrained by the poor nutrient quality of anthropogenic food (constraint hypothesis; see Introduction). In small passerines producing large clutches, the reserves required for egg production exceed what females can store endogenously (Perrins, 1996). Thus, they must acquire the nutrients for egg production daily from their diet when laying (Meijer & Drent, 1999). Following egg removal, urban females may have been unable to source sufficient high‐quality resources to invest in the production of new eggs. Previous results suggesting that the diet of urban blue tits is of lower quality than the diet of forest blue tits are in line with this explanation (Pollock et al., 2017).

Additionally, during the pre‐laying and laying periods, individuals divide energy between different activities that could trade off against egg production (Williams, 2005). For example, the energy costs associated with thermoregulation may limit the energy allocated to egg production (Martin & Wiebe, 2004). In great tits, the ability to replace eggs following egg removal is temperature constrained, with lower spring temperatures limiting egg production (Visser & Lessells, 2001). Low temperatures increase the daily energy expenditure of females during egg laying as they expend more energy on thermoregulation (Salvante et al., 2007). In our study, it is unlikely that temperature prevented urban birds from replacing eggs. Urban areas are generally 1–3°C warmer than surrounding natural areas due to the heat‐island effect (Kim, 1992). Thus, urban birds should spend less energy thermoregulating than forest birds due to the urban heat‐island.

Other processes apart from thermoregulation can impose resource allocation trade‐offs against egg production (Nager et al., 2001; Visser & Lessells, 2001). During laying, females have increased susceptibility to oxidative stress (Metcalfe & Monaghan, 2013), disease (Gustafsson et al., 1994) and parasite infection (Oppliger et al., 1997). It is possible that trade‐offs of this kind underlie the environmental constraints on egg laying in urban habitats. On the one hand, the nutrient requirements for the female's somatic maintenance may be fulfilled in the forest. Thus, forest females may invest any additional resources into egg production. On the other hand, as nutrient limitation may be higher in urban areas (Isaksson, 2018; Pollock et al., 2017; Senar et al., 2021), urban females in poor condition could be investing in somatic maintenance at the expense of egg quantity (Isaksson, 2015; Toledo et al., 2016). Thus, after egg removal, urban females may have insufficient nutrient reserves to form new eggs (Mänd et al., 2007).

### 4.2 Smaller clutch size in urban habitats as an adaptive response

Evidence suggests that urban populations of birds have smaller clutches than their non‐urban counterparts and our own data suggest that this is the case in our study population of blue tits (Branston et al., 2021). Additionally, previous research suggests that the number of nestlings that urban pairs can successfully rear is limited by food resources (Seress et al., 2020). It is, therefore, logical to pose whether the reduction in clutch size in urban populations is an adaptive strategy, where the number of eggs laid is optimised to match the (lower) number of nestlings that can successfully survive to fledging given the food resources in urban habitats. If this was the case, and in the absence of environmental constraints (see above), we predicted that urban females would lay new eggs following our egg removal manipulation to the same extent that forest females did so they can achieve the optimal clutch size for the habitat. Our data does not support this prediction. However, this interpretation should be taken cautiously since our study only took place in one year and among‐year variation in environmental conditions could affect the conclusions of our experiment.

It is also possible that smaller clutches in urban populations are explained by urban and non‐urban populations displaying contrasting life‐history strategies, where urban populations invest less in each reproductive attempt but live longer and breed through more years than individuals living in non‐urban environments (which would have fewer reproductive attempts across their shorter lives but invest more in each attempt). This idea is supported from studies that report higher winter survival in urban populations due to anthropogenic supplementary foods (Hanmer et al., 2022). However, it is still unclear how lifetime reproductive success compares between urban and non‐urban population of birds and whether urban and non‐urban populations are selected to have these two life‐history strategies.

In both habitats, our experimental manipulation led to a reduction in brood size, but this reduction was greater in the urban habitat since urban females laid fewer new eggs than forest females. Despite urban experimental nests having a smaller initial brood size than urban control nests, there was no difference in the number of offspring that fledged between the urban treatment groups. This was not the case in the forest, where control nests contained slightly more offspring than experimental nests across the brood‐rearing phase and at fledging. This contrasting result suggests that urban females may be laying more eggs than nestlings they can successfully rear (as urban control nests fledged proportionately fewer offspring when compared to [brood‐reduced] urban experimental nests). Given that traditional brood increase experiments have shown that birds can generally successfully rear more chicks than eggs they lay (Monaghan & Nager, 1997), our results suggest that the clutch size of urban females might not be optimally fine‐tuned to urban environmental conditions. It is possible that gene flow between the urban and forest populations might have homogenised gene pools, preventing the evolution of an adaptive urban clutch size (Postma & van Noordwijk, 2005) or that urban birds might be overestimating habitat quality when laying (Stracey & Robinson, 2012), for example, due to the high availability of low‐quality human‐provided resources (Broggi et al., 2022). Despite the lower productivity of urban breeding blue tits, there is little evidence to suggest that urban populations are declining. Therefore, urban blue tits must be compensating for their low productivity elsewhere. This could be mediated through life‐history trade‐offs, with urban blue tits investing less in each reproductive attempt but exhibiting a longer reproductive lifespan than their forest counterparts, with there being little difference in the fitness pay‐offs of birds breeding in these two habitats. Alternatively, urban populations could be supported by the continued dispersal of blue tits from higher productivity surrounding non‐urban habitats. In fact, this is supported by recent evidence which suggests that rural blue tit populations may supplement urban breeding populations (Hanmer et al., 2022).

### 4.3 The trade‐off between clutch size and egg volume

As the resources available to the breeding female are limited, individuals producing larger clutches may compensate by producing smaller eggs (Williams, 2001). We found that egg volume increased over the lay sequence for both urban treatment groups and for control clutches in the forest. In experimental forest nests, egg volume declined over the lay sequence, in line with previous studies suggesting that females have a limited ability to maintain egg quality when laying above their usual clutch size (Williams, 2005). In the urban habitat, nestlings hatched from smaller eggs may incur higher survival costs, and selection could be favouring females that can maintain, or increase, egg size across the lay sequence. Meanwhile, nestlings hatched from small eggs in forest habitats might be able to compensate for a small size at hatching in the nestling‐rearing phase (e.g. due to high‐quality food resources in forests). This may be important given the strong effect of weight on Day two (which is likely to be affected by egg volume; Krist, 2011) on nestling survival. If urban birds laid new eggs to the same extent as forest females following our manipulation, this could have detrimentally impacted egg and offspring quality, which they may be unable to compensate for during nestling‐rearing. Thus, in urban areas, females may terminate laying earlier than forest birds, before they pass their critical physiological threshold where egg size and clutch size are traded off against each other. Meanwhile, forest blue tits might be able to produce new eggs following egg removal, as they can likely compensate for any detrimental effects of egg size on offspring quality during the nestling‐rearing phase. This can be an alternative explanation for why urban females lay fewer new eggs than forest birds.

### 4.4 Limitations and future investigations

This study has limitations that could influence the interpretation of the results. First, we were unable to assess how parental quality and age affected the ability of females to respond to our experimental manipulation. Low‐quality or young individuals that naturally produce smaller clutches may be pushed into urban areas if free territories in the forest are no longer available (Isaksson, 2018). Indeed, laying following egg removal may be more common in forest habitats if females are more experienced at breeding (Oppliger et al., 1997). Second, this study consists of only one urban and one forest population, in a single  year. Replicated work is needed to assess if our results generalise across study populations and, importantly, how robust they are to among‐year variation in environmental conditions. Third, future work should compare the costs of each stage of the reproductive cycle (egg laying, incubation and nestling‐rearing) between urban and forest birds, and assess the consequences of these costs for clutch size optimisation, as there may be interactive effects between reproductive phases that operate differently between urban and forest habitats (Monaghan & Nager, 1997).

## 5 CONCLUSIONS

Our study provides initial experimental support for blue tits being more constrained to produce eggs in urban than forest habitats. Urban birds may experience greater energetic or nutrient constraints than forest birds that either immediately restricts egg formation or exacerbates the trade‐off between somatic maintenance and egg production in urban breeding females. Additionally, urban birds might produce small clutches as an increase in clutch size would be traded off against egg size, the detrimental effects of which birds might not be able to compensate for during the nestling‐rearing stage. The egg removal manipulation resulted in a brood reduction in the urban habitat, yet there was no difference in the number of offspring that fledged between the urban treatment groups. Thus, the clutch sizes of urban birds may be larger than what they could successfully rear, at least during the year of study. Overall, our results emphasise a need to incorporate the environmental constraints associated with egg production when attempting to explain variation in reproductive investment and success for birds breeding in anthropogenically modified landscapes.

## AUTHOR CONTRIBUTIONS

Mark D. Pitt, Pablo Capilla‐Lasheras, Davide M. Dominoni and Jelle J. Boonekamp conceived and designed the study. Mark D. Pitt, Pablo Capilla‐Lasheras, Claire J. Branston, Norah S. S. Alhowiti and Eugenio Carlon collected the data. Mark D. Pitt and Pablo Capilla‐Lasheras conducted the data analysis and wrote the first draft of the manuscript. All authors contributed to revise the manuscript.

## CONFLICT OF INTEREST STATEMENT

The authors declare no conflicts of interest.

## ETHICS STATEMENT

All work involving nest disturbance, egg removal and cross‐fostering were covered by the licence 207317 issued by NatureScot to Davide M. Dominoni. Permission for bird ringing was granted by the British Trust for Ornithology, with licences to Davide M. Dominoni (permit number: C6822) and Claire J. Branston (permit number: C6271).

## STATEMENT ON INCLUSION

Our study includes authors from numerous countries and backgrounds. All authors were approached early so that a diverse range of perspectives was considered prior to the study being conducted.

## Supporting information


**Appendix S1:** Supplementary information file. This file contains additional information on the methodologies used along with the outputs from each of the statistical models included in the main manuscript.

## Data Availability

Data available from the Dryad Digital Repository https://doi.org/10.5061/dryad.3j9kd51tf (Pitt et al., 2024).

## References

[jane14171-bib-0001] Andersson, M. N. , Wang, H. L. , Nord, A. , Salmón, P. , & Isaksson, C. (2015). Composition of physiologically important fatty acids in great tits differs between urban and rural populations on a seasonal basis. Frontiers in Ecology and Evolution, 3, 93.

[jane14171-bib-0002] Bates, D. , Kliegl, R. , Vasishth, S. , & Baayen, H. (2015). Parsimonious mixed models. *arXiv*, 1506.04967. 10.48550/arXiv.1506.04967

[jane14171-bib-0003] Biard, C. , Surai, P. F. , & Møller, A. P. (2005). Effects of carotenoid availability during laying on reproduction in the blue tit. Oecologia, 144(1), 32–44. 15868160 10.1007/s00442-005-0048-x

[jane14171-bib-0004] Branston, C. J. , Capilla‐Lasheras, P. , Pollock, C. J. , Griffiths, K. , White, S. , & Dominoni, D. M. (2021). Urbanisation weakens selection on the timing of breeding and clutch size in blue tits but not in great tits. Behavioural Ecology and Sociobiology, 75, 1–12.

[jane14171-bib-0005] Broggi, J. , Watson, H. , Nilsson, J. , & Nilsson, J. Å. (2022). Carry‐over effects on reproduction in food‐supplemented wintering great tits. Journal of Avian Biology, 2022(8), e02969.

[jane14171-bib-0006] Brooks, M. E. , Kristensen, K. , van Benthem, K. J. , Magnusson, A. , Berg, C. W. , Nielsen, A. , Skaug, H. J. , Machler, M. , & Bolker, B. M. (2017). glmmTMB balances speed and flexibility among packages for zero‐inflated generalized linear mixed modeling. The R Journal, 9(2), 378–400.

[jane14171-bib-0007] Capilla‐Lasheras, P. , Dominoni, D. M. , Babayan, S. A. , O'Shaughnessy, P. J. , Mladenova, M. , Woodford, L. , Pollock, C. J. , Barr, T. , Baldini, F. , & Helm, B. (2017). Elevated immune gene expression is associated with poor reproductive success of urban blue tits. Frontiers in Ecology and Evolution, 5, 64.

[jane14171-bib-0009] Capilla‐Lasheras, P. , Thompson, M. J. , Sánchez‐Tójar, A. , Haddou, Y. , Branston, C. J. , Réale, D. , Charmantier, A. , & Dominoni, D. M. (2022). A global meta‐analysis reveals higher variation in breeding phenology in urban birds than in their non‐urban neighbours. Ecology Letters, 25(11), 2552–2570. 36136999 10.1111/ele.14099 PMC9826320

[jane14171-bib-0010] Carey, C. (1996). Female reproductive energetics. In C. Carey (Ed.), Avian energetics and nutritional ecology (pp. 324–374). Springer US.

[jane14171-bib-0011] Chamberlain, D. E. , Cannon, A. R. , Toms, M. P. , Leech, D. I. , Hatchwell, B. J. , & Gaston, K. J. (2009). Avian productivity in urban landscapes: A review and meta‐analysis. Ibis, 151(1), 1–18.

[jane14171-bib-0012] Dorado‐Correa, A. M. , Rodríguez‐Rocha, M. , & Brumm, H. (2016). Anthropogenic noise, but not artificial light levels predicts song behaviour in an equatorial bird. Royal Society Open Science, 3(7), 160231. 27493778 10.1098/rsos.160231 PMC4968470

[jane14171-bib-0013] Fehlmann, G. , O'riain, M. J. , Fürtbauer, I. , & King, A. J. (2021). Behavioral causes, ecological consequences, and management challenges associated with wildlife foraging in human‐modified landscapes. BioScience, 71(1), 40–54. 33442328 10.1093/biosci/biaa129 PMC7791362

[jane14171-bib-0014] Fenoglio, M. S. , Calviño, A. , González, E. , Salvo, A. , & Videla, M. (2021). Urbanisation drivers and underlying mechanisms of terrestrial insect diversity loss in cities. Ecological Entomology, 46(4), 757–771.

[jane14171-bib-0015] Gao, J. , & O'Neill, B. C. (2020). Mapping global urban land for the 21st century with data‐driven simulations and shared socioeconomic pathways. Nature Communications, 11(1), 2302. 10.1038/s41467-020-15788-7 PMC7210308 32385275

[jane14171-bib-0016] Glądalski, M. , Bańbura, M. , Kaliński, A. , Markowski, M. , Skwarska, J. , Wawrzyniak, J. , Zieliński, P. , Cyżewska, I. , & Bańbura, J. (2017). Differences in the breeding success of blue tits Cyanistes caeruleus between a forest and an urban area: A long‐term study. Acta Ornithologica, 52(1), 59–68.

[jane14171-bib-0018] Graveland, J. , & Drent, R. H. (1997). Calcium availability limits breeding success of passerines on poor soils. Journal of Animal Ecology, 66, 279–288.

[jane14171-bib-0019] Gustafsson, L. , Nordling, D. , Andersson, M. S. , Sheldon, B. C. , & Qvarnström, A. (1994). Infectious diseases, reproductive effort and the cost of reproduction in birds. Philosophical Transactions of the Royal Society of London B: Biological Sciences, 346(1317), 323–331. 7708827 10.1098/rstb.1994.0149

[jane14171-bib-0020] Hanmer, H. J. , Dadam, D. , & Siriwardena, G. M. (2022). Evidence that rural wintering bird populations supplement suburban breeding populations. Bird Study, 69(1–2), 12–27.

[jane14171-bib-0021] Haywood, S. (1993a). Role of extrinsic factors in the control of clutch‐size in the blue tit *Parus caeruleus* . Ibis, 135(1), 79–84.

[jane14171-bib-0022] Haywood, S. (1993b). Sensory and hormonal control of clutch size in birds. The Quarterly Review of Biology, 68(1), 33–60.

[jane14171-bib-0023] Iannone, R. , Cheng, J. , Schloerke, B. , & Hughes, E. (2023). Seo J. gt: Easily create presentation‐ready display table . R package version 0.8. 0. 2022.

[jane14171-bib-0024] Isaksson, C. (2015). Urbanization, oxidative stress and inflammation: A question of evolving, acclimatizing or coping with urban environmental stress. Functional Ecology, 29(7), 913–923.

[jane14171-bib-0025] Isaksson, C. (2018). Impact of urbanization on birds. Bird Species, 235, 257.

[jane14171-bib-0026] Jarrett, C. , Powell, L. L. , McDevitt, H. , Helm, B. , & Welch, A. J. (2020). Bitter fruits of hard labour: Diet metabarcoding and telemetry reveal that urban songbirds travel further for lower‐quality food. Oecologia, 193(2), 377–388. 32533359 10.1007/s00442-020-04678-w PMC7320956

[jane14171-bib-0027] Jensen, J. K. , Jayousi, S. , von Post, M. , Isaksson, C. , & Persson, A. S. (2022). Contrasting effects of tree origin and urbanization on invertebrate abundance and tree phenology. Ecological Applications, 32(2), e2491. 34757670 10.1002/eap.2491

[jane14171-bib-0029] Kim, H. H. (1992). Urban heat Island. International Journal of Remote Sensing, 13(12), 2319–2336.

[jane14171-bib-0030] Krist, M. (2011). Egg size and offspring quality: A meta‐analysis in birds. Biological Reviews, 86(3), 692–716. 21070586 10.1111/j.1469-185X.2010.00166.x

[jane14171-bib-0031] Lack, D. (1947). The significance of clutch‐size. Ibis, 89(2), 302–352.

[jane14171-bib-0032] Lack, D. (1954). The natural regulation of animal numbers. Clarendon Press.

[jane14171-bib-0033] Liu, X. , Hu, G. , Chen, Y. , Li, X. , Xu, X. , Li, S. , Pei, F. , & Wang, S. (2018). High‐resolution multi‐temporal mapping of global urban land using Landsat images based on the Google Earth Engine Platform. Remote Sensing of Environment, 209, 227–239.

[jane14171-bib-0034] Lüdecke, D. , Ben‐Shachar, M. S. , Patil, I. , Waggoner, P. , & Makowski, D. (2021). performance: An R package for assessment, comparison and testing of statistical models. Journal of Open Source Software, 6(60), 3139.

[jane14171-bib-0035] Mänd, R. , Tilgar, V. , Kilgas, P. , & Mägi, M. (2007). Manipulation of laying effort reveals habitat‐specific variation in egg production constraints in great tits (*Parus major*). Journal of Ornithology, 148, 91–97.

[jane14171-bib-0036] Martin, K. , & Wiebe, K. L. (2004). Coping mechanisms of alpine and arctic breeding birds: Extreme weather and limitations to reproductive resilience. Integrative and Comparative Biology, 44(2), 177–185. 21680497 10.1093/icb/44.2.177

[jane14171-bib-0037] McKinney, M. L. (2008). Effects of urbanization on species richness: A review of plants and animals. Urban Ecosystems, 11, 161–176.

[jane14171-bib-0038] McMaster, D. G. , Neudorf, D. L. , Sealy, S. G. , & Pitcher, T. E. (2004). A comparative analysis of laying times in passerine birds. Journal of Field Ornithology, 75(2), 113–122.

[jane14171-bib-0039] Meijer, T. , & Drent, R. (1999). Re‐examination of the capital and income dichotomy in breeding birds. Ibis, 141(3), 399–414.

[jane14171-bib-0040] Metcalfe, N. B. , & Monaghan, P. (2013). Does reproduction cause oxidative stress? An open question. Trends in Ecology & Evolution, 28(6), 347–350. 23485157 10.1016/j.tree.2013.01.015

[jane14171-bib-0041] Monaghan, P. , & Nager, R. G. (1997). Why don't birds lay more eggs? Trends in Ecology & Evolution, 12(7), 270–274. 21238065 10.1016/s0169-5347(97)01094-x

[jane14171-bib-0042] Nager, R. G. , Monaghan, P. , & Houston, D. C. (2001). The cost of egg production: Increased egg production reduces future fitness in gulls. Journal of Avian Biology, 32(2), 159–166.

[jane14171-bib-0043] Nicolaus, M. , Michler, S. P. , Ubels, R. , van der Velde, M. , Komdeur, J. , Both, C. , & Tinbergen, J. M. (2009). Sex‐specific effects of altered competition on nestling growth and survival: An experimental manipulation of brood size and sex ratio. Journal of Animal Ecology, 78(2), 414–426. 19054223 10.1111/j.1365-2656.2008.01505.x

[jane14171-bib-0045] Oppliger, A. , Christe, P. , & Richner, H. (1997). Clutch size and malarial parasites in female great tits. Behavioral Ecology, 8(2), 148–152.

[jane14171-bib-0046] Perrins, C. M. (1996). Eggs, egg formation and the timing of breeding. Ibis, 138(1), 2–15.

[jane14171-bib-0047] Pitt, M. D. , Alhowiti, N. S. , Branston, C. J. , Carlon, E. , Boonekamp, J. J. , Dominoni, D. M. , & Capilla‐Lasheras, P. (2024). Data from: Environmental constraints can explain clutch size differences between urban and forest blue tits: Insights from an egg removal experiment. *Dryad Digital Repository*, 10.5061/dryad.3j9kd51tf PMC11880655 39219149

[jane14171-bib-0048] Pollock, C. J. , Capilla‐Lasheras, P. , McGill, R. A. , Helm, B. , & Dominoni, D. M. (2017). Integrated behavioural and stable isotope data reveal altered diet linked to low breeding success in urban‐dwelling blue tits (*Cyanistes caeruleus*). Scientific Reports, 7(1), 5014. 28694437 10.1038/s41598-017-04575-y PMC5503996

[jane14171-bib-0049] Postma, E. , & van Noordwijk, A. J. (2005). Gene flow maintains a large genetic difference in clutch size at a small spatial scale. Nature, 433(7021), 65–68. 15635410 10.1038/nature03083

[jane14171-bib-0050] R Core Team . (2022). R: A language and environment for statistical computing. Vienna, Austria.

[jane14171-bib-0051] Salvante, K. G. , Walzem, R. L. , & Williams, T. D. (2007). What comes first, the zebra finch or the egg: Temperature‐dependent reproductive, physiological and behavioural plasticity in egg‐laying zebra finches. Journal of Experimental Biology, 210(8), 1325–1334. 17401116 10.1242/jeb.02745

[jane14171-bib-0052] Senar, J. C. , Manzanilla, A. , & Mazzoni, D. (2021). A comparison of the diet of urban and forest great tits in a Mediterranean habitat. Animal Biodiversity and Conservation, 44(2), 321–327.

[jane14171-bib-0053] Sepp, T. , McGraw, K. J. , Kaasik, A. , & Giraudeau, M. (2018). A review of urban impacts on avian life‐history evolution: Does city living lead to slower pace of life? Global Change Biology, 24(4), 1452–1469. 29168281 10.1111/gcb.13969

[jane14171-bib-0054] Seress, G. , Sándor, K. , Evans, K. L. , & Liker, A. (2020). Food availability limits avian reproduction in the city: An experimental study on great tits *Parus major* . Journal of Animal Ecology, 89(7), 1570–1580. 32419138 10.1111/1365-2656.13211

[jane14171-bib-0055] Shmueli, G. , Minka, T. P. , Kadane, J. B. , Borle, S. , & Boatwright, P. (2005). A useful distribution for fitting discrete data: Revival of the Conway–Maxwell–Poisson distribution. Journal of the Royal Statistical Society Series C: Applied Statistics, 54(1), 127–142.

[jane14171-bib-0056] Sjoberg, D. D. , Whiting, K. , Curry, M. , Lavery, J. A. , & Larmarange, J. (2021). Reproducible summary tables with the gtsummary package. The R Journal, 13(1), 570–580.

[jane14171-bib-0060] Stenning, M. (2018). The blue tit. Bloomsbury Publishing.

[jane14171-bib-0061] Stoffel, M. A. , Nakagawa, S. , & Schielzeth, H. (2017). rptR: Repeatability estimation and variance decomposition by generalized linear mixed‐effects models. Methods in Ecology and Evolution, 8(11), 1639–1644.

[jane14171-bib-0062] Stracey, C. M. , & Robinson, S. K. (2012). Are urban habitats ecological traps for a native songbird? Season‐long productivity, apparent survival, and site fidelity in urban and rural habitats. Journal of Avian Biology, 43(1), 50–60.

[jane14171-bib-0063] Toledo, A. , Andersson, M. N. , Wang, H. L. , Salmón, P. , Watson, H. , Burdge, G. C. , & Isaksson, C. (2016). Fatty acid profiles of great tit (*Parus major*) eggs differ between urban and rural habitats, but not between coniferous and deciduous forests. The Science of Nature, 103, 1–11. 10.1007/s00114-016-1381-0 PMC4908168 27300022

[jane14171-bib-0064] Troscianko, J. (2014). A simple tool for calculating egg shape, volume and surface area from digital images. Ibis, 156(4), 874–878.

[jane14171-bib-0065] van Bohemen, H. D. (1998). Habitat fragmentation, infrastructure and ecological engineering. Ecological Engineering, 11, 199–207.

[jane14171-bib-0066] Visser, M. E. , & Lessells, C. M. (2001). The costs of egg production and incubation in great tits (*Parus major*). Proceedings of the Royal Society of London B: Biological Sciences, 268(1473), 1271–1277. 10.1098/rspb.2001.1661 PMC1088737 11410154

[jane14171-bib-0068] Williams, T. D. (2001). Experimental manipulation of female reproduction reveals an intraspecific egg size clutch size trade‐off. Proceedings of the Royal Society of London B: Biological Sciences, 268(1465), 423–428. 10.1098/rspb.2000.1374 PMC1088623 11270440

[jane14171-bib-0069] Williams, T. D. (2005). Mechanisms underlying the costs of egg production. BioScience, 55(1), 39–48.

[jane14171-bib-0070] Womack, R. J. , Capilla‐Lasheras, P. , McGlade, C. L. , Dominoni, D. M. , & Helm, B. (2023). Reproductive fitness is associated with female chronotype in a songbird. Animal Behaviour, 205, 65–78.

