# Supplementary material for: Environmental constraints can explain clutch size differences between urban and forest blue tits: Insights from an egg removal experiment
Source: J Anim Ecol. 2024 Sep 1;94(3):368–78. doi: 10.1111/1365-2656.14171 (PMC11880655; doi:10.1111/1365-2656.14171)
Supplement: Supplementary file 1 — Appendix S1: Supplementary information file. This file contains additional information on the methodologies used along with the outputs from each of the statistical models included in the main manuscript. [file JANE-94-368-s001.docx]

**Supplementary material**

***Supplementary methods***

***Cross-fostering procedure***

We did experimental crosses between habitats as well as control within habitat crosses, resulting in 16 and 14 broods cross fostered between habitats in the forest and urban environment respectively; and 12 and six broods cross fostered within habitats in the forest and urban environment respectively (sample sizes vary across habitats due to low nestling survival in the urban habitat). Nests were paired either across habitats or within habitats based on hatching date and half broods were swapped across paired nests (for odd brood sizes, we swapped half the brood size minus one). Nestlings were transported in artificial nests made of cotton wool and kept inside thermal boxes including one heat pack. Since nestlings cannot be ringed at this young age, we marked individual nestlings with a combination of two non-permanent and non-toxic colour markers.

***Repeatability of the egg volume measurements***

We used the R package *rptR* *v.0.9.22* (Stoffel *et al*., 2017) to quantify the repeatability of the volume measurements calculated in IMAGEJ. To estimate repeatability, we used a mixed effects model framework, including the fixed effect structure that we specify in the main text for egg volume analysis. Clutch ID (a 59-level factor) and egg ID (386-level factor) were included as random effect intercepts. Repeatability was calculated for egg ID (consistency of the volume measurements per egg) and clutch ID (consistency of the volume measurements per clutch [i.e., nest-box], equivalent to how consistent egg size was within an individual female). For experimental nests, where the first four eggs laid were weighed, we used Pearson’s correlation coefficient *r* to test the strength of the relationship between egg mass and egg volume.

***Nestling body mass on days six and 12 after hatching***

We investigated whether nestling body mass (in grams) later in the nestling-rearing phase depended on the treatment group and habitat. We created two separate linear mixed models, one for weight six days after hatching and one for weight 12 days after hatching. These models included as fixed effect predictors: the number of siblings alive at day two (mean-centred), hatch date (mean-centred), time of day of weight measurement (morning *versus* afternoon), habitat of hatching, habitat of rearing, treatment group and the two-way interaction between habitat of rearing and treatment group, and habitat of hatching and treatment group. We also included the clutch ID of hatching (a 55-level factor) and the clutch ID of rearing (a 51-level factor) as random effect intercepts in both models on body mass.

**Supplementary results**

***Effect of treatment group and urban environment on nestling body mass six days after hatching***

Egg removal had no effect on nestling weight on day 6, either in the interaction with habitat of rearing or habitat of hatching, or as a single effect predictor (Table S7). Habitat of rearing had a strong effect on nestling weight six days after hatching, with nestlings reared in the urban environment being 0.643 grams lighter than same-age nestlings reared in the forest (Table S7). Habitat of hatching, time of the day, number of siblings in the nest on day 2 and hatching date did not explain variation in nestling weight six days after hatching (Table S7).

***Effect of treatment group and urban environment on nestling body mass 12 days after hatching***

Egg removal had no effect on nestling weight on day 12, either in the interaction with habitat of rearing or habitat of hatching, or as a single effect predictor (Table S8). Habitat of rearing had a strong effect on nestling weight 12 days after hatching, with nestlings reared in the urban environment being 1.50 grams lighter than same-age nestlings reared in the forest (Table S8). Early hatched nestlings were also heavier on day 12 that those nestlings hatched later in the season (Table S8). Habitat of hatching, time of the day and number of siblings in the nest on day 2 did not explain variation in nestling weight 12 days after hatching (Table S8).

**Supplementary tables**

**Table S1**. Summary of vegetation in the two study locations, Kelvingrove Park (44532.18 m^2^ surveyed; coordinates: 55.869, −4.2851) and SCENE (113804.46 m^2^; coordinates: 56.129,−4.6145). For methodological details, see Branston *et al.*, 2021.

| **Location** | **Tree Genus** | **Trees / m^2^** |
| --- | --- | --- |
| Kelvingrove Park | *Acer* | 0.00184 |
|  | *Quercus* | 0.00076 |
|  | *Fraxinus* | 0.00074 |
|  | *Ilex* | 0.00067 |
|  | *Betula* | 0.00052 |
|  | *Ulmus* | 0.00052 |
|  | *Salix* | 0.00036 |
|  | *Crataegus* | 0.00029 |
|  | *Tilia* | 0.00029 |
|  | Others | 0.00236 |
| SCENE | *Quercus* | 0.00899 |
|  | *Betula* | 0.00076 |
|  | *Sorbus* | 0.00022 |
|  | *Ilex* | 0.00019 |
|  | *Prunus* | 0.00014 |
|  | *Pinus* | 0.00012 |
|  | *Alnus* | 0.00006 |
|  | *Fraxinus* | 0.00002 |
|  | *Picea* | 0.00002 |
|  | Others | 0.00003 |

**Table S2.** Coefficients and likelihood-ratio tests of Conway-Maxwell Poisson model explaining variation in the total of number of eggs laid by blue tit females (n = 61 broods, 34 broods in the forest and 27 broods in the urban habitat). The quadratic term for first egg laying date did not receive statistical support (χ^2^_1_ = 0.01, p = 0.980) and was dropped from the full model to ease interpretation of single effect predictors. Model coefficients are shown in the link-function scale (‘log’).

| **Fixed effect** | **Estimate** | **SE***^A^* | **95% CI***^A^* | **χ^2^** | **df*^A^*** | **p** | |
| --- | --- | --- | --- | --- | --- | --- | --- |
| **Intercept** | 2.231 | 0.040 | 2.153, 2.309 |  |  |  | |
| **Treatment group** |  |  |  |  |  |  | |
| *Egg removal* | 0.167 | 0.055 | 0.059, 0.275 |  |  |  | |
| **Habitat** |  |  |  |  |  |  | |
| *Urban* | -0.135 | 0.065 | -0.261, -0.008 |  |  |  | |
| **First egg laying date** | -0.016 | 0.005 | -0.026, -0.006 | 9.68 | 1 | 0.002 | |
| **Treatment group × Habitat** |  |  |  | 1.37 | 1 | 0.243 | |
| *Egg removal × Urban* | -0.105 | 0.089 | -0.280, 0.070 |  |  |  | |
| *^A^* SE = Standard Error, CI = Confidence Interval, df = degrees of freedom likelihood-ratio test | | | | | | |  |

**Table S3.** Coefficients and likelihood-ratio tests of Conway-Maxwell Poisson model explaining variation in the total of number of eggs laid by forest blue tit females (n = 34 broods). Model coefficients are shown in the link-function scale (‘log’).

| **Fixed effect** | **Estimate** | **SE***^A^* | **95% CI***^A^* | **χ^2^** | **df*^A^*** | **p** | |
| --- | --- | --- | --- | --- | --- | --- | --- |
| **Intercept** | 2.256 | 0.040 | 2.178, 2.333 |  |  |  | |
| **Treatment group** |  |  |  | 7.70 | 1 | 0.006 | |
| *Egg removal* | 0.163 | 0.056 | 0.054, 0.273 |  |  |  | |
| **First egg laying date** | -0.019 | 0.007 | -0.033, -0.005 | 6.80 | 1 | 0.009 | |
| *^A^* SE = Standard Error, CI = Confidence Interval, df = degrees of freedom likelihood-ratio test. | | | | | | |  |

**Table S4.** Coefficients and likelihood-ratio tests of Conway-Maxwell Poisson model explaining variation in the total of number of eggs laid by urban blue tit females (n = 27 broods). Model coefficients are shown in the link-function scale (‘log’).

| **Fixed effect** | **Estimate** | **SE*^A^*** | **95% CI*^A^*** | **χ^2^** | **df*^A^*** | **p** |  |
| --- | --- | --- | --- | --- | --- | --- | --- |
| **Intercept** | 2.070 | 0.050 | 1.971, 2.168 |  |  |  |  |
| **Treatment group** |  |  |  | 0.71 | 1 | 0.398 |  |
| *Egg removal* | 0.059 | 0.069 | -0.077, 0.194 |  |  |  |  |
| **First egg laying date** | -0.013 | 0.007 | -0.027, 0.001 | 3.11 | 1 | 0.078 |  |
| *^A^* SE = Standard Error, CI = Confidence Interval, df = degrees of freedom likelihood-ratio test. | | | | | | | |

**Table S5.** Coefficients and likelihood-ratio tests of Gaussian linear mixed model explaining variation in egg volume (mm^3^) (n = 386 eggs from 59 clutches). The quadratic term for first egg laying date did not receive statistical support (χ^2^_1_ = 0.862, p = 0.353) and was dropped from the full model to ease interpretation of single effect predictors.

| **Fixed effect** | **Estimate** | **SE*^A^*** | **95% CI*^A^*** | **χ^2^** | **df*^A^*** | **p** |
| --- | --- | --- | --- | --- | --- | --- |
| **Intercept** | 1,280.289 | 33.619 | 1,214.396, 1,346.182 |  |  |  |
| **Laying order** | 8.512 | 4.239 | 0.204, 16.820 |  |  |  |
| **Treatment group** |  |  |  |  |  |  |
| *Egg removal* | 79.121 | 44.533 | -8.163, 166.405 |  |  |  |
| **Habitat** |  |  |  |  |  |  |
| *Urban* | 27.851 | 50.634 | -71.390, 127.093 |  |  |  |
| **Number of eggs laid** | -13.989 | 8.350 | -30.355, 2.377 | 2.93 | 1 | 0.087 |
| **Egg laying date** | -9.958 | 2.715 | -15.280, -4.636 | 13.17 | 1 | **<** 0.001 |
| **Laying order × Treatment group** |  |  |  |  |  |  |
| *Laying order ×*  *Egg removal* | -13.691 | 4.675 | -22.853, -4.529 |  |  |  |
| **Laying order × Habitat** |  |  |  |  |  |  |
| *Laying order × Urban* | 3.479 | 5.895 | -8.075, 15.034 |  |  |  |
| **Treatment group × Habitat** |  |  |  |  |  |  |
| *Egg removal × urban* | -50.408 | 67.868 | -183.426, 82.610 |  |  |  |
| **Laying order × Treatment group × Habitat** |  |  |  | 3.94 | 1 | 0.047 |
| *Laying order ×*  *Egg removal ×*  *Urban* | 15.559 | 7.786 | 0.298, 30.820 |  |  |  |
| *^A^* SE = Standard Error, CI = Confidence Interval, df = degrees of freedom likelihood-ratio. | | | | | | |

**Table S6.** Coefficients and likelihood-ratio tests of Gaussian linear mixed model explaining variation in 2-day-old nestling weight (grams) (n = 346 nestlings from 50 clutches). The quadratic term for hatching date (χ^2^_1_ = 0.07, p = 0.793) and the 2-way interaction between habitat and treatment group (χ^2^_2_ = 0.03, p = 0.857) did not receive statistical support and were dropped from the full model to ease interpretation of single effect predictors.

| **Fixed effect** | **Estimate** | **SE*^A^*** | **95% CI*^A^*** | **χ^2^** | **df*^A^*** | **p** | |
| --- | --- | --- | --- | --- | --- | --- | --- |
| **Intercept** | 2.146 | 0.126 | 1.898, 2.394 |  |  |  | |
| **Treatment group** |  |  |  | 2.91 | 1 | 0.088 | |
| *Egg removal* | -0.211 | 0.130 | -0.466, 0.044 |  |  |  | |
| **Habitat** |  |  |  | 2.56 | 1 | 0.109 | |
| *Urban* | -0.193 | 0.128 | -0.443, 0.057 |  |  |  | |
| **Time of the day** |  |  |  | 0.51 | 1 | 0.477 | |
| *Morning* | -0.077 | 0.121 | -0.315, 0.161 |  |  |  | |
| **Number of siblings** | -0.023 | 0.030 | -0.081, 0.035 | 0.63 | 1 | 0.427 | |
| **Hatching date** | 0.013 | 0.016 | -0.018, 0.045 | 0.69 | 1 | 0.407 | |
| *^A^* SE = Standard Error, CI = Confidence Interval, df = degrees of freedom likelihood-ratio. | | | | | | |  |

**Table S7.** Coefficients and likelihood-ratio tests of Gaussian linear mixed model explaining variation in 6-day-old nestling weight (grams) (n = 338 nestlings). The quadratic term for hatching date (χ^2^_1_ = 0.05, p = 0.829) and the 2-way interactions between habitat of rearing and treatment group (χ^2^_2_ = 0.25, p = 0.617) and habitat of hatching and treatment group (χ^2^_1_ = 0.01, p = 0.926) did not receive statistical support and were dropped from the full model to ease interpretation of single effect predictors.

| **Fixed effect** | **Estimate** | **SE*^A^*** | **95% CI*^A^*** | **χ^2^** | **df*^A^*** | **p** | |
| --- | --- | --- | --- | --- | --- | --- | --- |
| **Intercept** | 6.005 | 0.261 | 5.493, 6.517 |  |  |  | |
| **Treatment group** |  |  |  | 0.05 | 1 | 0.819 | |
| *Egg removal* | 0.060 | 0.281 | -0.491, 0.612 |  |  |  | |
| **Habitat of rearing** |  |  |  | 5.80 | 1 | 0.016 | |
| *Urban* | -0.643 | 0.268 | -1.169, -0.117 |  |  |  | |
| **Habitat of hatching** |  |  |  | 3.16 | 1 | 0.076 | |
| *Urban* | -0.439 | 0.252 | -0.932, 0.054 |  |  |  | |
| **Time of the day** |  |  |  | 0.44 | 1 | 0.508 | |
| *Morning* | -0.157 | 0.250 | -0.648, 0.334 |  |  |  | |
| **Number of siblings** | -0.090 | 0.058 | -0.204, 0.025 | 2.59 | 1 | 0.107 | |
| **Hatching date** | -0.004 | 0.035 | -0.072, 0.064 | 0.01 | 1 | 0.911 | |
| *^A^* SE = Standard Error, CI = Confidence Interval, df = degrees of freedom likelihood-ratio. | | | | | | |  |

**Table S8.** Coefficients and likelihood-ratio tests of Gaussian linear mixed model explaining variation in 12-day-old nestling weight (grams) (n = 273 nestlings). The quadratic term for hatching date (χ^2^_1_ = 1.04, p = 0.309) and the 2-way interactions between habitat of rearing and treatment group (χ^2^_2_ = 0.41, p = 0.524) and habitat of hatching and treatment group (χ^2^_1_ = 0.80, p = 0.371) did not receive statistical support and were dropped from the full model to ease interpretation of single effect predictors.

| **Fixed effect** | **Estimate** | **SE*^A^*** | **95% CI*^A^*** | **χ^2^** | **df*^A^*** | **p** |
| --- | --- | --- | --- | --- | --- | --- |
| **Intercept** | 10.698 | 0.285 | 10.138, 11.257 |  |  |  |
| **Treatment group** |  |  |  | 1.11 | 1 | 0.291 |
| *Egg removal* | 0.295 | 0.291 | -0.275, 0.865 |  |  |  |
| **Habitat of rearing** |  |  |  | 20.15 | 1 | **<** 0.001 |
| *Urban* | -1.500 | 0.324 | -2.134, -0.866 |  |  |  |
| **Habitat of hatching** |  |  |  | 0.09 | 1 | 0.760 |
| *Urban* | -0.074 | 0.255 | -0.574, 0.425 |  |  |  |
| **Time of the day** |  |  |  | 2.00 | 1 | 0.157 |
| *Morning* | -0.370 | 0.279 | -0.916, 0.177 |  |  |  |
| **Number of siblings** | -0.046 | 0.059 | -0.162, 0.070 | 0.65 | 1 | 0.420 |
| **Hatching date** | -0.099 | 0.036 | -0.169,  -0.028 | 7.82 | 1 | 0.005 |
| *^A^* SE = Standard Error, CI = Confidence Interval, df = degrees of freedom likelihood-ratio. | | | | | |  |

**Table S9.** Coefficients and likelihood-ratio tests of Binomial linear mixed model explaining variation in the number of nestlings alive per brood at different time points through nestling development (n = 48 broods). The quadratic term for hatching date (χ^2^_1_ = 0.02, p = 0.879), the 3-way interaction between habitat, nestling age and treatment group (χ^2^_3_ = 5.23, p = 0.156), and the 2-way interaction between habitat and treatment group (χ^2^_1_ = 0.07, p = 0.794) did not receive statistical support and were dropped from the full model to ease interpretation of other predictors.

| **Fixed effect** | **Estimate** | **SE*^A^*** | **95% CI*^A^*** | **χ^2^** | **df*^A^*** | | **p** | | |
| --- | --- | --- | --- | --- | --- | --- | --- | --- | --- |
| **Intercept** | 3.144 | 0.568 | 2.031, 4.257 |  |  |  | |  |  |
| **Treatment group** |  |  |  |  |  |  | |  |  |
| *Egg removal* | 1.402 | 0.753 | -0.073, 2.877 |  |  |  | |  |  |
| **Habitat** |  |  |  |  |  |  | |  |  |
| *Urban* | -0.836 | 0.711 | -2.230, 0.558 |  |  |  | |  |  |
| **Time point** |  |  |  |  |  |  | |  |  |
| *Day6* | -0.014 | 0.434 | -0.865, 0.837 |  |  |  | |  |  |
| *Day13* | -0.406 | 0.415 | -1.220, 0.407 |  |  |  | |  |  |
| *Fledged* | -1.142 | 0.392 | -1.911,  -0.373 |  |  |  | |  |  |
| **Hatching date** | -0.119 | 0.091 | -0.297, 0.060 | 1.70 | 1 | 0.193 | |  |  |
| **Treatment group × Time point** |  |  |  | 8.69 | 3 | 0.034 | |  |  |
| *Egg removal* × Day6 | -1.672 | 0.606 | -2.861,  -0.483 |  |  |  | |  |  |
| *Egg removal* × Day12 | -0.765 | 0.605 | -1.951, 0.422 |  |  |  | |  |  |
| *Egg removal* × Fledged | -1.066 | 0.603 | -2.247, 0.115 |  |  |  | |  |  |
| **Habitat × Time point** |  |  |  | 32.09 | 3 | < 0.001 | |  |  |
| *Urban × Day6* | -0.997 | 0.544 | -2.063, 0.069 |  |  |  | |  |  |
| *Urban × Day13* | -2.570 | 0.548 | -3.644,  -1.496 |  |  |  | |  |  |
| *Urban ×* *Fledged* | -2.532 | 0.546 | -3.603,  -1.461 |  |  |  | |  |  |
| *^A^* SE = Standard Error, CI = Confidence Interval, df = degrees of freedom likelihood-ratio. | | | | | | | | |  |

**Table S10.** Coefficients and likelihood-ratio tests of Binomial linear mixed model explaining variation in individual nestling survival to fledging (n = 315 nestlings). The quadratic term for hatching date (χ^2^_1_ = 1.82, p = 0.178) and the 2-way interaction between habitat of rearing and treatment group (χ^2^_1_ = 0.40, p = 0.527) did not receive statistical support and were dropped from the full model to ease interpretation of other predictors.

| **Fixed effect** | **Estimate** | **SE*^A^*** | **95% CI*^A^*** | **χ^2^** | **df*^A^*** | **p** |  |
| --- | --- | --- | --- | --- | --- | --- | --- |
| **Intercept** | 2.116 | 2.320 | -2.432, 6.663 |  |  | 0.362 |  |
| **Treatment group** |  |  |  | 0.02 | 1 | 0.902 |  |
| *Egg removal* | 0.255 | 2.083 | -3.828, 4.338 |  |  |  |  |
| **Habitat of hatching** |  |  |  | 0.89 | 1 | **0.346** |  |
| *Urban* | 1.651 | 1.674 | -1.631, 4.933 |  |  |  |  |
| **Habitat of rearing** |  |  |  | 21.22 | 1 | **< 0.001** |  |
| *Urban* | -12.032 | 3.828 | -19.536,  -4.528 |  |  |  |  |
| **Hatching weight (day 2)** | 2.788 | 0.984 | 0.860, 4.717 | 11.57 | 1 | **0.001** |  |
| **Hatching date** | -0.628 | 0.347 | -1.309, 0.053 | 2.91 | 1 | **0.088** |  |
| *^A^* SE = Standard Error, CI = Confidence Interval | | | | | | | |

**References in Supplementary materials**

Branston, C.J., Capilla-Lasheras, P., Pollock, C.J., Griffiths, K., White, S. and Dominoni, D.M., 2021. Urbanisation weakens selection on the timing of breeding and clutch size in blue tits but not in great tits. *Behavioral Ecology and Sociobiology*, *75*, pp.1-12. Available at: <https://doi.org/10.1007/s00265-021-03096-z>.

Stoffel, M.A., Nakagawa, S. and Schielzeth, H., 2017. rptR: Repeatability estimation and variance decomposition by generalized linear mixed‐effects models. *Methods in ecology and evolution*, *8*(11), pp.1639-1644. Available at: [**https://doi.org/10.1111/2041-210X.12797**](https://doi.org/10.1111/2041-210X.12797).
